# Tungsten Ditelluride: a layered semimetal

**DOI:** 10.1038/srep10013

**Published:** 2015-06-12

**Authors:** Chia-Hui Lee, Eduardo Cruz Silva, Lazaro Calderin, Minh An T. Nguyen, Matthew J. Hollander, Brian Bersch, Thomas E. Mallouk, Joshua A. Robinson

**Affiliations:** 1 Department of Materials Science and Engineering, The Pennsylvania State University, University Park, Pennsylvania 16802, United States; 2 Center for 2-Dimensional and Layered Materials, The Pennsylvania State University, University Park, Pennsylvania 16802, United States; 3 Materials Research Institute, The Pennsylvania State University, University Park, United States; 4 Department of Physics, The Pennsylvania State University, University Park, Pennsylvania 16802, United States; 5 Research Computing and Cyberinfrastructure, The Pennsylvania State University, University Park, Pennsylvania 16802, United States; 6 Department of Chemistry, and Department of Biochemistry and Molecular Biology, The Pennsylvania State University, University Park, Pennsylvania 16802, United States; 7 Department of Electrical Engineering, The Pennsylvania University, University Park, Pennsylvania 16802, United States

## Abstract

Tungsten ditelluride (WTe_2_) is a transition metal dichalcogenide (TMD) with physical and electronic properties that make it attractive for a variety of electronic applications. Although WTe_2_ has been studied for decades, its structure and electronic properties have only recently been correctly described. We experimentally and theoretically investigate the structure, dynamics and electronic properties of WTe_2_, and verify that WTe_2_ has its minimum energy configuration in a distorted 1T structure (Td structure), which results in metallic-like transport. Our findings unambiguously confirm the metallic nature of WTe_2_, introduce new information about the Raman modes of Td-WTe_2_, and demonstrate that Td-WTe_2_ is readily oxidized via environmental exposure. Finally, these findings confirm that, in its thermodynamically favored Td form, the utilization of WTe_2_ in electronic device architectures such as field effect transistors may need to be reevaluated.

The rise of graphene was a defining point for the discovery and development of stable two-dimensional layered materials (2DLM)^1^,^2^. This breakthrough has stimulated the exploration of 2D materials such as hexagonal boron nitride (hBN)^3^ and transition-metal dichalcogenides (TMDs)^4^ of formula MX_2_, where M is a IVB-VIB transition metal atom (IVB: Ti and Zr; V-B: Nb and Ta; VI-B: Mo and W) and X is a chalcogen (S, Se, or Te). Due to the *d*-orbitals involved in their electronic structure, the TMDs exhibit a wide range of electronic properties that have led to advances in practical devices, including field effect transistors^5^,^6^,^7^,^8^,^9^,^10^,^11^,^12^, photodetectors^13^,^14^, chemical^15^ and biosensors^16^,^17^,^18^, and nano-electromechanical systems (NEMS)^19^,^20^.

The synthesis, structure, and electronic properties of TMD systems have been recently explored using both experimental and theoretical techniques^4^,^21^,^22^,^23^,^24^,^25^,^26^,^27^. Among the many attributes, the absence of dangling bonds and interface traps^22^, as well as the presence of finite and distinctive band gaps^5^,^22^,^28^, make TMDs attractive as components of tunneling field effect transistors (TFETs). The foundation of the TFET is to combine dissimilar TMDs in such a way that electrons in the valence band of layers of VIB-TMDs can easily tunnel into the conduction band of layers of IVB-TMDs. Among these materials, single-layer 2H-WTe_2_ is expected to have the narrowest band gap of the semiconducting VIB-TMDs at ~0.7 eV^5^,^23^,^29^,^30^. This suggests a high electron mobility that could maximize the efficiency of electron injection in TMD TFETs^9^,^12^.

The physical and electronic properties of WTe_2_ and other TMDs have been widely modeled for their device performance as semiconductor materials based on a 2H structure^11^,^21^,^23^,^26^,^29^,^30^,^31^,^32^,^33^, despite the fact that experimental data and non-constrained geometry optimization using density functional theory (DFT) provide strong evidence that WTe_2_ has its lowest energy in a distorted 1T (Td) structure^34^, also called 1T’^35^,^36^. Both 2H^37^,^38^ and Td structures^35^,^39^,^40^,^41^,^42^,^43^,^44^ have been reported in experimental studies of WTe_2_, and recent reports have focused on the extraordinary magnetoresistance^35^,^36^,^45^ and thermoelectric^32^,^45^ properties of Td-WTe_2_. In this paper, we study bulk and exfoliated, crystalline WTe_2_ synthesized under near equilibrium conditions. WTe_2_ is found to have a distorted 1T structure and semimetallic electronic characteristics. The results presented here agree with early theory and experiments from the 1960s^34^,^39^,^40^,^43^, unlike several recent theoretical calculations and predictions of WTe_2_ based on a MoS_2_-like trigonal prismatic isomorph^23^,^26^,^29^,^30^,^31^,^32^,^46^.

## Results and Discussion

**Figure 1:**
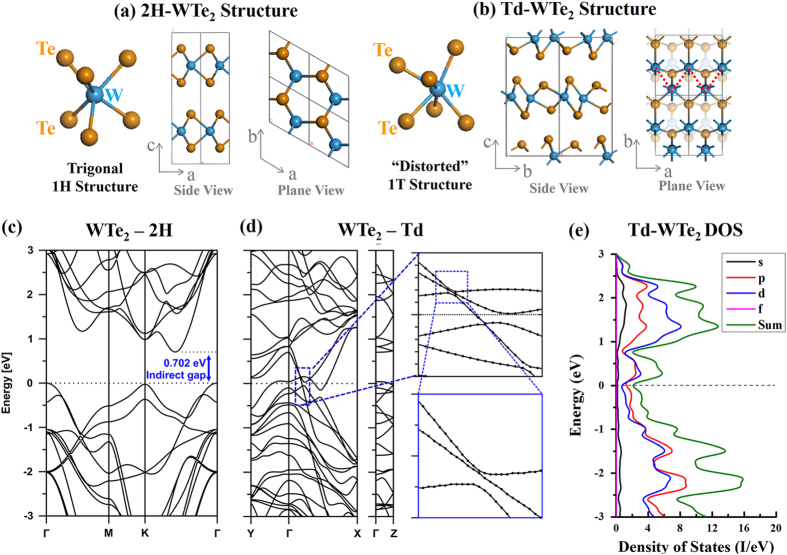
Comparison of the tungsten-tellurium coordination (side and plane views) of (**a**) 2H-WTe_2_ and (**b**) distorted “1T”, or Td-WTe_2_. The theoretical 2H structure exhibits trigonal prismatic coordination with uniformly displaced atoms, whereas tungsten atoms in the Td structure are octahedrally coordinated by Te with alternating long and short distances between W atoms due to strong intermetallic bonding. The electronic band structures (**c**) indicate that bulk WTe_2_ in the 2H structure has an indirect 0.702 eV bandgap. Bulk WTe_2_ in the Td structure (**d**) has a 0.21 eV band overlap in Γ-X, and the density of states (**e**) reaches a minimum, but never goes to zero near Fermi level.

To verify the stable phase and electronic properties of WTe_2_, we utilize DFT to model the crystal structures, based on atomic positions calculated from X-ray diffraction patterns of Td-WTe_2_^44^ and from a hypothetical 2H-WTe_2_ structure^11^,^23^,^26^,^29^,^47^ (Fig. 1). The 2H-WTe_2_ structure (Fig. 1a) has hexagonal symmetry. The upper and lower tellurium atoms are bonded to a central W atom, forming a trigonal prismatic arrangement similar to that found in 2H-MoS_2_ and 2H-WSe_2_^21^,^48^. The Td-WTe_2_ structure (Fig. 1b) is similar to that of the 1T polytype, in which the upper tellurium atoms are rotated by 180^o^ with respect to the lower tellurium atoms, forming W-centered octahedra. However, in Td-WTe_2_ the tungsten atoms are shifted by 0.87 Å in the layer plane and 0.15 Å in the perpendicular direction (along the c-axis) from the center of the octahedron. This shift of tungsten atoms results from a shortened metal-metal distance in transition metal tellurides^49^ due to strong intermetallic bonding^34^,^50^. As a consequence, the tungsten atoms are unequally spaced and form a zigzag chain along the a-axis (Fig. 1b). The distances between tungsten atoms in Td-WTe_2_ alternate along the b-axis at 2.862 and 4.394 Å, in contrast to the 2H phase where they are equally spaced at a distance of 3.6 Å. Additionally, the tellurium atoms are no longer coplanar, but instead exhibit a zigzag structure with 0.6 Å c-axis variation in atomic positions. Finally, the tungsten–tellurium bond lengths are also uneven at 2.719 and 2.815 Å, compared to a uniform 2.769 Å for 2H-WTe_2_. A detailed comparison of the 2H-WTe_2_ and Td-WTe_2_ crystal structures, lattice parameters, and bond angles are given in the Supplementary Information. Adjacent WTe_2_ layers exhibit AB stacking, where each layer is rotated 180^o^ with respect to each other. These changes in bonding environment result in the lowering of the lattice symmetry from hexagonal to orthorhombic. Since WTe_2_ layers are bound by weak van der Waals interaction, we also performed a structural optimization using the Grimme method for van der Waals corrections^51^,^52^, but it was observed that LDA yields a better description of the stacking distance, as summarized in Table S2 of the supplementary information.

The crystal structure plays a significant role in the characteristic electronic properties of WTe_2_. Based on the optimized structures of 2H- and Td-WTe_2_, we have calculated the electronic band structures and summarized the results in Fig. 1. Full band structures are displayed in Figure S2 in the supplementary information. Fig. 1c shows the band structure for 2H-WTe_2_. The *d*-orbitals of tungsten split into three different bands and the 2H-WTe_2_ trigonal prismatic coordination gives rise to a calculated 0.702 eV bandgap. In contrast, the band structure and low density of states (DOS) at the Fermi energy of bulk Td-WTe_2_ (Fig. 1d) shows that it is a semimetal, with few bands crossing the Fermi energy in the three main axes of the Brillouin zone. The highest valence band bends upward while the lowest conduction band bends downward to form a 0.21 eV overlap, confirming the findings of Augustin *et al.*^34^ A detailed calculation of the band structure around the crossing point in the ΓX segment shows an indirect band overlap of 0.3 eV, with a separation of 11 meV among the bands at their closest point (see inset in Fig. 1d), which is well below the thermal energy at room temperature (25 meV).

**Figure 2:**
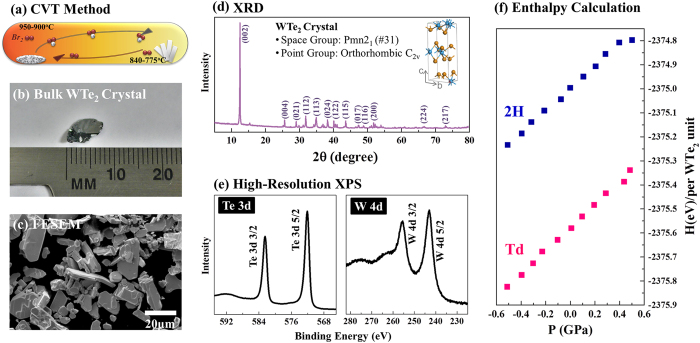
Synthesis of WTe_2_ by (**a**) chemical vapor transport results in the formation of (**b**) large bulk crystals, accompanied by (**c**) smaller crystallites. X-ray powder diffraction (**d**) confirms that WTe_2_ crystallizes in space group Pmn2_1_ in the Td structure. (**e**) XPS shows that Td-WTe_2_ is stoichiometric. Density functional theory (**f**) indicates that the enthalpy of formation of the Td-WTe_2_ phase is lower than that of the 2H phase, regardless of pressure, indicating it is the most stable form of bulk Td-WTe_2_.

Bulk WTe_2_ crystals were grown by chemical vapor transport (CVT) as in previous reports (Fig. 2a),^34^,^40^,^41^,^42^,^44^,^50^ using bromine (Br) as the transport agent. Following the synthesis, the powder and bulk crystals were characterized by X-ray powder diffraction (XRD), scanning electron microscopy (SEM), X-ray photoelectron spectroscopy (XPS), Raman spectroscopy, and temperature-dependent current-voltage (I-V) measurements. SEM shows that bulk WTe_2_ crystals (Fig. 2b) exhibit a platelet morphology (Fig. 2c) with no apparent angles that would be expected for hexagonal crystals. XRD patterns (Fig. 2d) indicates an atomic arrangement based on the primitive orthorhombic space group Pmn2_1_, consistent with the formation of the Td structure. The experimental XRD pattern collected from bulk WTe_2_ crystal was compared to simulated XRD patterns based on the Td-WTe_2_ and 2H-WTe_2_ structures, and shown in Figure S3 in supplementary information. It can be observed that distinctive reflections of the Td structure are present in the experimental diffractogram. Moreover, W:Te ratio calculated from high-resolution elemental XPS spectra from W 4d and Te 3d regions in Fig. 2e confirms a W:Te ratio of 1:1.9 for bulk WTe_2_ crystals, suggesting a slight Te deficiency. The full range XPS in Figure S4 also confirms the W:Te ratio and verifies that the transport agent is not incorporated in significant quantities into the WTe_2_. To understand why the Td structure is favored, the enthalpies of formation of 2H and Td-WTe_2_ were calculated within a pressure range near equilibrium, which is representative of the chemical vapor transport (CVT) method (Fig. 2f). As is evident from Fig. 2f, the enthalpy of formation for the Td structure is lower at equilibrium (zero pressure) by 0.58 eV per WTe_2_ formula unit. This is also the case for non-equilibrium synthesis conditions up to at least 0.6 GPa.

**Figure 3:**
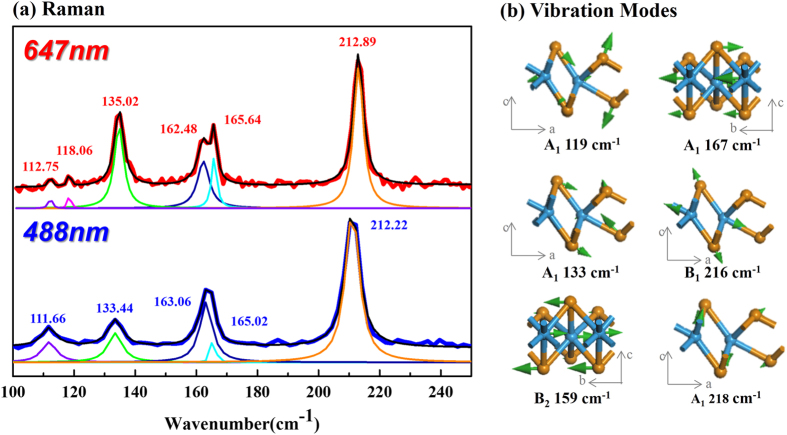
(**a**) Raman spectra of Td-WTe_2_ using 647 (red) and 488 nm (blue) laser excitation. The peaks in the spectra can be assigned to in-plane and out-of-plane (**b**) Raman-active vibrational modes. The major peaks were processed with Lorentzian peak fitting in both spectra, and the two in-plane vibrational modes at 162 and 167 cm^−1^ were deconvoluted in the spectrum obtained with 647 nm excitation.

To date, there are no reports on the vibrational properties of 2H- or Td-WTe_2_. We have explored the vibrational properties as a function of incident photon energy via Raman spectroscopy, and the results are shown in Fig. 3. Flakes of Td-WTe_2_ were exfoliated onto SiO_2_/Si substrates (each>10 layers thick), and Raman spectra were acquired using 647 and 488 nm laser excitations. With 488 nm excitation, the vibrational modes are dominated by peaks at 112, 133, 163, 165, and 212 cm^−1^. The same vibrational modes are evident with 647 nm excitation, but with slight frequency shifts, and an additional peak appears at 118 cm^−1^. To understand the origin of the Raman peaks, we used density functional perturbation theory (DFPT)^53^ to calculate the vibrational modes in Td-WTe_2_. The calculated modes, as well as the symmetry analysis and their infrared and Raman activity are listed in Table S3 (Supplementary Information), and the phonon modes that correlate to the experimentally observed vibrations (Fig. 3a) are shown in Fig. 3b. Because of the structural distortion induced by metal-metal bonding, the out-of-plane vibrational modes of Td-WTe_2_ are not oriented perpendicular to the WTe_2_ sheets. The out-of-plane Raman-active modes for Td-WTe_2_ are vibrating either along the W-Te bond or at an angle to the vertical line, in contrast to the A_1g_ mode of 2H-WTe_2_ that involves atomic motion perpendicular to the layer plane. The 118, 133, and 212 cm^−1^ peaks in the Td-WTe_2_ Raman spectrum are identified as “tilted” out-of-plane A_1_ modes at 119, 133, 218 cm^−1^ and a tilted B_1_ vibrational mode at 216 cm^−1^. The two deconvoluted peaks near 163 cm^−1^ and 166 cm^−1^ in the 647 nm Raman spectrum in Fig. 3(a) are identified as the in-plane B_2_ and A_1_ vibrational modes in different directions; the observed frequencies are close to the calculated 159 and 167 cm^−1^ frequencies in the model. The other calculated Raman-active modes in Table S3 may be too low in intensity (relative to the background signal) to be observed in the Raman scattering experiments.

**Figure 4:**
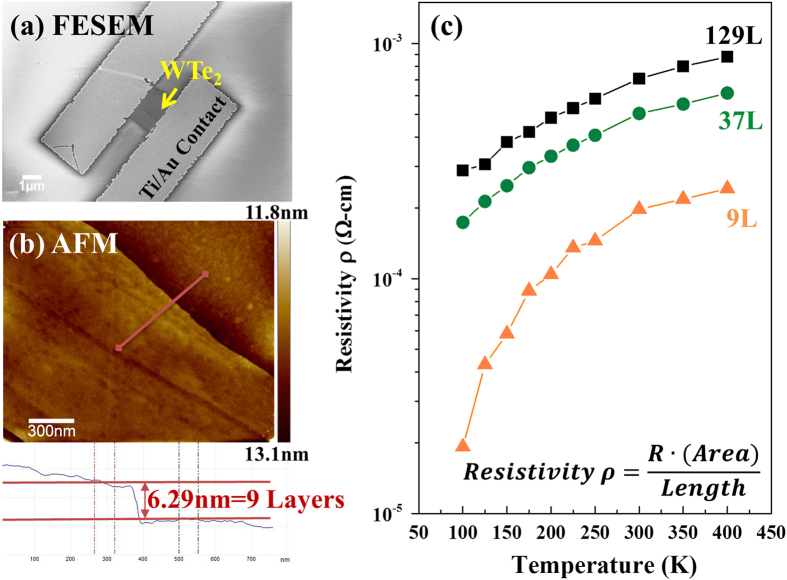
Representative (**a**) SEM and (**b**) AFM images of 9-layer (9L) Td-WTe_2_ Resistivity measurements as a function of temperature (**c**) confirm that Td-WTe_2_ is metallic in nature (resistivity proportional to temperature), rather than the semiconducting behavior recently suggested^22^,^28^,^29^,^30^.

Temperature dependent resistance measurements confirm the metallic nature of synthetic Td-WTe_2_. This is verified for thick (9 - 130 layers, measured by AFM) exfoliated Td-WTe_2_ flakes. Two-terminal device structures were fabricated using titanium-gold electrodes (Fig. 4a). The series contact resistance was found to be 6.76 × 10^−5^ Ω cm using a transmission line measurement (TLM),^54^ and was subtracted from the total measured resistance. Figure 4c shows the temperature-dependent resistivity, which varies between 1 × 10^−3^ and 7 × 10^−3^ Ω cm at 300 K, depending on the layer thickness. The different values obtained at different layer thicknesses suggest that the layer structure may affect carrier transport though Td-WTe_2_. Importantly, the resistivity of WTe_2_ is strongly correlated to temperature, increasing with increasing temperature over most of the range measured. The positive temperature dependence of the resistivity and the bulk resistivity values, which are ~2 orders of magnitude higher than those of ordinary metals at 300 K^55^, are consistent with the calculation that Td-WTe_2_ is metallic in nature. We note that while two-terminal measurements do not provide direct access to the carrier concentration, and therefore confirmation of semi-metallic WTe_2_, they are sufficient to verify that Td-WTe_2_ is not semiconducting – a critical point for the device community when considering this material in electronic device architectures.

**Figure 5:**
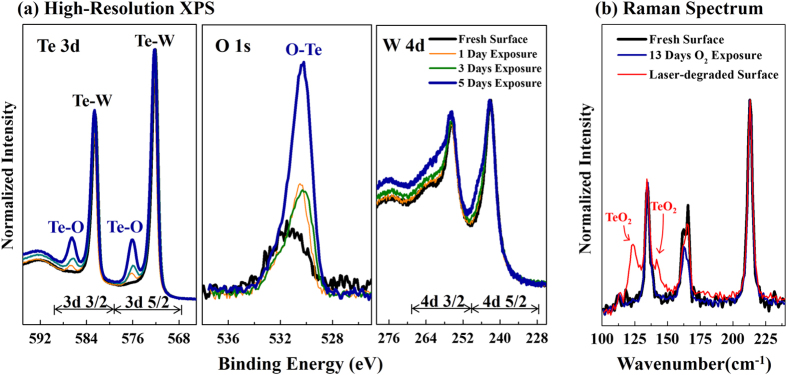
Stability testing of WTe_2_ flakes in ambient air by (**a**) high-resolution elemental XPS spectra and (**b**) Raman spectroscopy. XPS reveals an increase in Te-O bonding in the Td 3d and O 1s spectra with minor changes in the W 4d spectra, indicating the formation of TeO_2_ on the surface. The Raman spectrum after 13 days of air exposure (blue) shows small changes in intensity of the two in-plane modes in the 160-167 cm^−1^ region. The Raman spectrum of a laser-degraded sample (red) suggests that photon-assisted oxidation can lead to rapid degradation of WTe_2_ via the formation of TeO_2_.

Finally, the stability of WTe_2_ is a critical aspect of robust operation in a variety of applications. In the case of exfoliated flakes, the Raman spectra evolved with time during the data collection process, indicating that environmental sensitivity must be considered. Surface characterization tools such as XPS and Raman spectroscopy were used to understand surface stability and sensitivity to ambient conditions. Figure 5 summarizes the high-resolution XPS and Raman spectra, which compare fresh exfoliated WTe_2_ with WTe_2_ that was exposed to ambient (air, 1 atmosphere, room temperature) conditions for extended periods of time. The XPS peak positions of the fresh exfoliated and aged WTe_2_ surfaces are listed in Table S4. Each XPS spectrum was calibrated with the carbon C 1s binding energy (BE) position and corrected with a relative sensitivity factor (R.S.F.). For the high resolution elemental XPS spectrum, normalization of intensities was used to compare spectra collected from the same exfoliated WTe_2_ sample with increasing exposure time to air. Elemental XPS analysis reveals the evolution of a secondary chemical bond in the Te 3d peaks corresponding to an increase in Te-O binding. The primary degradation appears to be the formation of Te-O bonds, which is accompanied by an increase in the intensity of the O 1s peak, and formation of a small energy loss peak at the left shoulder of the W 4d region. This indicates that the WTe_2_ surface is air sensitive, which could affect the stability of few-layer exfoliated WTe_2_. Table S4 lists the binding energies from peak fitting analysis of the Te 3d, O1s and W 4d regions of the spectra of WTe_2_ and degraded (or oxidized) WTe_2_. There are two sets of Te 3d3/2 and Te 3d5/2 binding energies from the peak fitting analysis, which refer to the Te 3d binding energies of the fresh exfoliated WTe_2_ surface and those of TeO_2_ from the NIST XPS database^60^,^61^. Raman spectra in Fig. 5b show that the aged WTe_2_ surface may have minor changes near the 162-167 cm^−1^ region of in-plane vibrational modes. However, the Raman spectra may not be sensitive enough to detect the formation of tellurium oxides on the surface. With laser excitation, using the 647 nm laser and three periods of 45 seconds acquisition time, the WTe_2_ surface is visibly modified and two new vibrational modes at 124 and 142 cm^−1^ were detected. These peak positions correlate well with those of TeO_2_, and confirm the formation of Te-O bonds under accelerated aging, suggesting this as the mechanism of degradation for Td-WTe_2_ when exposed to air or a combination of photons and air^52^.

## Conclusion

The distorted 1T structure (Td) of bulk tungsten ditelluride has been experimentally verified to be thermodynamically stable relative to the 2H polymorph. The calculated band structure of Td-WTe_2_ shows a 0.21 eV indirect band overlap from the Γ to X direction, indicating that it is a semimetallic TMD material. Raman spectra and DFT simulations provide evidence that the out-of-plane vibrational modes involve atomic motions at angles that are displaced from the c-axis direction because the distorted octahedral bonding in Td-WTe_2_. We have experimentally verified that Td-WTe_2_ behaves as a metal, with an as-yet unexplained strong dependence of the resistivity on the thickness of multilayer flakes.

We have also evaluated the stability of thin flakes (9 – 130 layers) and found that care must be taken to ensure that oxidation does not occur, as the surface of Td-WTe_2_ is sensitive to ambient air. Finally, we note that this work clearly verifies that WTe_2_, grown via CVT under near equilibrium conditions, is not a semiconductor. This ultimately requires careful reconsideration of the use of WTe_2_ in a variety of device^22^,^28^ architectures.

## Methods

### Crystal Growth

Tungsten ditelluride (WTe_2_) bulk crystals were produced by the chemical vapor transport (CVT) method with bromine as the transport agent. WTe_2_ powder was synthesized by heating a mixture containing stoichiometric amounts of tungsten (Acros Organics 99.9%) and tellurium (Strem Chemicals 99.9%) at 800 °C for 3 days in an evacuated and sealed quartz ampoule (10 mm ID, 12 mm OD, 150 mm length). The mixture was slowly heated from room temperature to 800 °C for 12 h; slow heating was used to minimize the possibility of explosion due to the strong exothermicity of the reaction. Some tellurium sublimed into the cooler zone of the ampoule (~350 ^o^C), so the two ends of the ampoule were kept at 950 °C and 775 °C for another day to ensure that all the tellurium reacted with the tungsten. WTe_2_ single crystals were grown from the synthesized powder by chemical vapor transport with bromine (Sigma-Aldrich, 99.8 + %) as the transport gas at ~6 mg/cm^3^. The growth process ran for 4 d in an evacuated and sealed quartz ampoule (10 mm ID, 12 mm OD, 100 mm length), the hot and growth zones of which were kept at 840 °C and 900 °C, respectively. The resulting crystals were pumped under dynamic vacuum at room temperature for 1 d in order to remove any residual bromine.

### Mechanical Exfoliation

WTe_2_ flakes were mechanically exfoliated onto fresh and cleaned Si/SiO_2_ substrates via the “scotch-tape” method^53^ and imaged by using an Olympus MX50 optical microscope.

### Characterization

WTe_2_ powder and crystals were analyzed by X-ray powder diffraction (XRD) using a PANalytical XPert Pro MPD theta-theta diffractometer with Cu α x-ray source. Energy dispersive spectroscopy (EDS) on a FEI Nova NanoSEM 630 FESEM as well as Kratos Analytical Axis Ultra X-ray photoelectron spectra (XPS) by Kratos Analytical Axis Ultra were used to confirm the stoichiometry of both WTe_2_ powders and bulk crystals, and investigate the surface bonding and stability of the WTe_2_ flakes. Raman spectroscopy of exfoliated thick and few-layer WTe_2_ flakes was carried out using a Renishaw inVia confocal microscope-based Raman spectrometer with a spectral resolution less than 1 cm^−1^. Laser power was kept at 0.2mW at all times with 488 and 647 nm laser excitations. Electrical properties of WTe_2_ samples of different thicknesses were tested using two Ti/Au contacts made by a lift-off process at both edges of the exfoliated WTe_2_ flakes. Total resistance measurements were collected by using a Lakeshore Cryo Probe Station, which controlled the temperature from liquid nitrogen temperature 77 K to 400 K under vacuum. The size of each flake was measured by using images obtained with a Leo 1530 Field Emission Scanning Electron Microscope (FESEM) operated at 2 kV. The thickness and number of layers of the WTe_2_ flakes were determined by atomic force microscopy (AFM) using a Bruker Dimension Icon in tapping mode in air.

### Theoretical calculations

A Td-WTe_2_ crystal structure model was created from the crystallographic data reported by B. Brown^49^ in 1966, adjusting the axes to match the conventional Pmn2_1_ representation; while the 2H model was constructed from a MoS_2_ based model with lattice parameters from Kumar *et al.*^29^ and Ding *et al.*^26^ Geometry optimization of the initial structures was followed by the calculation of their electronic structures, vibrational properties, as well as enthalpies of formation as function of pressure, within Density Functional Theory (DFT), as implemented in CASTEP^56^ (Materials Studio 6.1, Accelrys, accelrys.com), as well as in Quantum Espresso 5.1.^57^ The Local Density Approximation (LDA) as parameterized by Perdew and Zunger^58^,^59^ was selected for exchange and correlation functional, and dispersion corrections were implemented following the semi-empirical Grimme method (LDA + DFT-D)^51^,^52^. Norm-conserving pseudopotentials were used for all the elements. Convergence analysis for the total energy, band gaps and forces set the cutoff energy of the plane wave basis set at 740 eV (CASTEP) and 680 eV (QE), and a Monkhorst-Pack grid of 10x20 × 5 for sampling of the Brillouin zone. Under these computational conditions the total energy and band gaps were converged to 0.1 meV. Geometrical optimizations were performed for both the LDA and LDA plus DFT-D functions until the structures reached configurations with energy differences of 5 × 10^−6^ eV/atom, and forces were less than 0.01 eV/A.

## Additional Information

**How to cite this article**: Lee, C.-H. *et al.* Tungsten Ditelluride: a layered semimetal. *Sci. Rep.* **5**, 10013; doi: 10.1038/srep10013 (2015).

## Supplementary Material

Supplementary Infomration
