# Supplementary material for: Tungsten Ditelluride: a layered semimetal
Source: Sci Rep. 2015 Jun 12;5:10013. doi: 10.1038/srep10013 (PMC5155493; doi:10.1038/srep10013)
Supplement: Supplementary Infomration [file srep10013-s1.pdf]

Supplementary Information

Tungsten ditelluride: a layered semimetal

Chia-Hui Lee [a,b,c], Eduardo Cruz Silva [b,d], Lazaro Calderin [c,e], Minh An T. Nguyen [d,f], Matthew J. Hollander [g], Brian Bersch [a,b,c], Thomas E. Mallouk [d,f], Joshua A. Robinson [a,b,c,]*

[a.] Department of Materials Science and Engineering, The Pennsylvania State University, University Park, Pennsylvania 16802, United States, [b.] Center for 2-Dimensional and Layered Materials, The Pennsylvania State University, University Park, Pennsylvania 16802, United States, [c.] Materials Research Institute, The Pennsylvania State University, University Park, United States, [d.] Department of Physics, The Pennsylvania State University, University Park, Pennsylvania 16802, United States, [e.] Research Computing and Cyberinfrastructure, The Pennsylvania State University, University Park, Pennsylvania 16802, United States, [f.] Department of Chemistry, and Department of Biochemistry and Molecular Biology, The Pennsylvania State University, University Park, Pennsylvania 16802, United States, [g.] Department of Electrical Engineering, The Pennsylvania University, University Park, Pennsylvania 16802, United States.

**Corresponding Author: Joshua A. Robinson (jrobinson@psu.edu)*

[*] Author correspondence: jrobinson@psu.edu

Crystal data and unit cell parameters of the 2H- and Td-WTe_2 initial crystal structures are listed in Table S1. The initial coordinates of the tungsten and tellurium atoms in the 2H- and Td-WTe_2 unit cells are also included in Table S1.

Table S1. 2H- and Td-WTe_2 crystal structures.

	2H Structure[1,2]			Td Structure[3]		
**Crystal System**	Hexagonal			Orthorhombic		
**Space Group**	$P6_3/mmc$			$Pmn2_1$		
**Point Group**	D_{6h}			C_{2v}		
	**Unit Cell Parameters**					
	a(Å)	b(Å)	c(Å)	a(Å)	b(Å)	c(Å)
	3.60	3.60	14.18	3.50	6.28	14.07

**Atoms**	**Initial Coordinates**					
	u	v	w	u	v	W
**W 1**	0.3333	0.6667	0.2500	0.5000	0.9005	0.0000
**W 2**	0.6667	0.3333	0.7500	0.0000	0.0995	0.5000
**W 3**		n/a		0.0000	0.5414	0.9851
**W 4**		n/a		0.5000	0.4586	0.4851
**Te 1**	0.3333	0.6667	0.6210	0.5000	0.2941	0.0965
**Te 2**	0.6667	0.3333	0.1210	0.5000	0.8517	0.3893
**Te 3**	0.6667	0.3333	0.3790	0.0000	0.7059	0.5965
**Te 4**	0.3333	0.6667	0.8790	0.0000	0.1483	0.8893
**Te 5**		n/a		0.0000	0.8002	0.1400
**Te 6**		n/a		0.0000	0.3559	0.3449
**Te 7**		n/a		0.5000	0.1998	0.6400
**Te 8**		n/a		0.5000	0.6441	0.8449

The optimized WTe_2 lattice parameters and bandgap information calculated by LDA and LDA plus DFT-D methods are listed in Table S2. The relaxed LDA coordinates of the tungsten and tellurium atoms in the 2H- and Td-WTe_2 unit cells are also included in Table S2.

Table S2. DFT Calculation results of WTe_2 in different crystal structures

**WTe_2 Structures**	**2H**				**Td**			
**Bulk Model**	**Lattice Parameters**			**Bandgap,**	**Lattice Parameters**			**Bandgap,**
	**a**	**b**	**c**	**Eg(eV)**	**a**	**b**	**c**	**Eg(eV)**
**LDA CA-PZ**	3.49	3.49	13.78	0.706	3.46	6.24	13.73	0
**LDA+DFT-D**	3.47	3.47	13.61	0.630	3.46	6.25	13.43	0

**Atoms**	**Relaxed Coordinates**					
	u	v	w	u	v	w
**W 1**	0.3333	0.6667	0.2500	0.5000	0.8998	0.0007
**W 2**	0.6667	0.3333	0.7500	0.0000	0.1002	0.5007
**W 3**		n/a		0.0000	0.5414	0.9845
**W 4**		n/a		0.5000	0.4586	0.4845
**Te 1**	0.3333	0.6667	0.6190	0.5000	0.2936	0.09645
**Te 2**	0.6667	0.3333	0.1190	0.5000	0.8526	0.3889
**Te 3**	0.6667	0.3333	0.3810	0.0000	0.7074	0.5964
**Te 4**	0.3333	0.6667	0.8810	0.0000	0.1474	0.8889
**Te 5**		n/a		0.0000	0.7976	0.1433
**Te 6**		n/a		0.0000	0.3547	0.3419
**Te 7**		n/a		0.5000	0.2024	0.6433
**Te 8**		n/a		0.5000	0.6453	0.8419

Te-W-Te angles within the Te layers are 75.7° and 83.3° for the upper Te layer and 81.9° and 80.2° for the lower Te layer; the analogous bond angle is 81.1° in 2H-WTe_2. The upper Te-W-lower Te bond angles are 77.4°, 97.8° and 116.1° compared to equivalent 82.7° angles in the 2H structure.

(a) 2H-WTe_2 Structure **(b) Td-WTe_2 Structure**

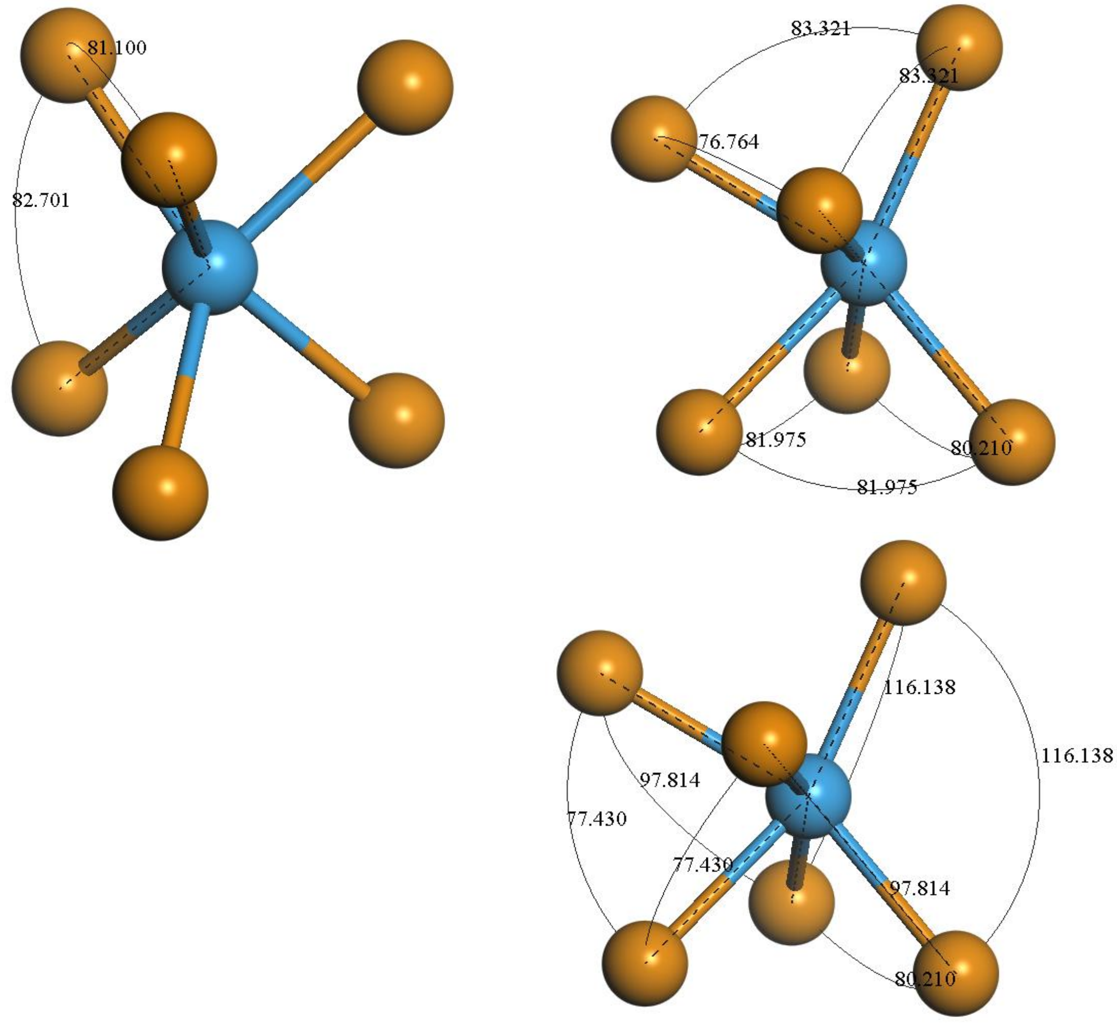

Figure S1. Te-W-Te bond angles in the (a) 2H-WTe_2 and (b) Td-WTe_2 structures.

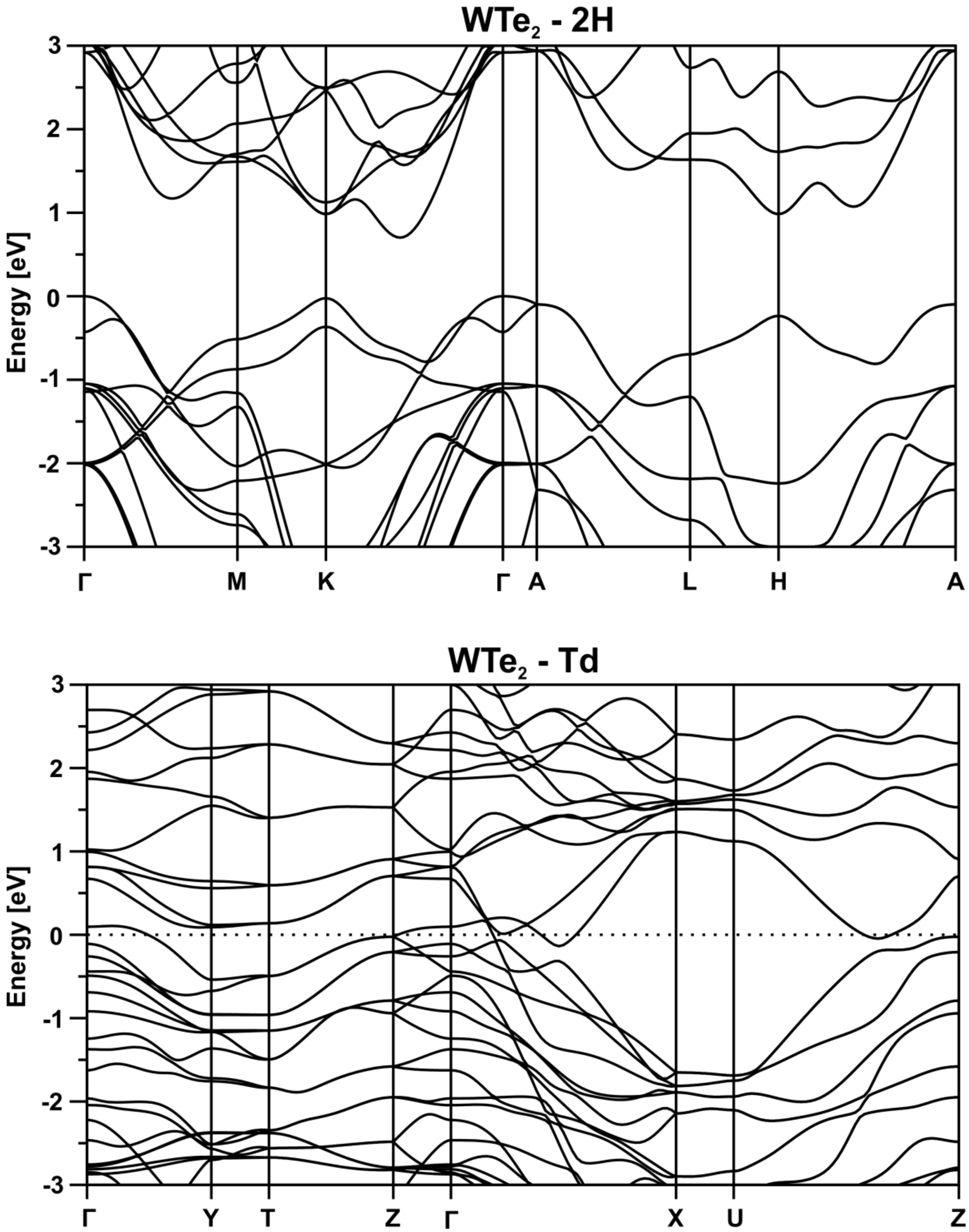

Figure S2. Full electronic band structure for the hexagonal (2H) phase (top), and the distorted tetragonal (Td) phase (bottom) of WTe_2.

We have analyzed our WTe_2 experimental x-ray diffraction (XRD) patterns with the existed WTe_2 diffraction patterns PDF#04-007-0799 in International Centre for Diffraction Data (ICDD) database, and confirmed the bulk WTe_2 single crystal is orthorhombic crystal system. We have also compared our experimental XRD patterns with the theoretical XRD patterns of Td-WTe_2 and 2H-WTe_2 in Figure S3.

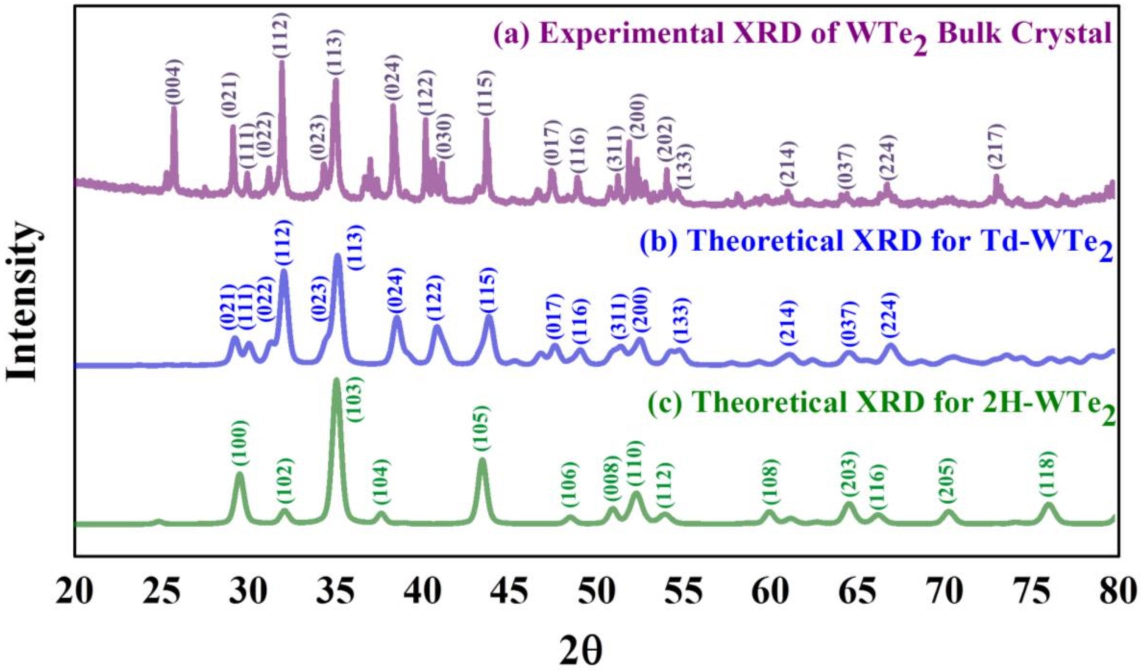

Figure S3. X-ray Diffraction Patterns for (a) experimental WTe_2 bulk crystal; (b) theoretical Td-WTe_2; (c) theoretical 2H-WTe_2.

The phonon frequencies and symmetry analysis of IR and Raman activities were calculated with DFPT, and are listed in Table S3.

Table S3. Calculated vibrational modes, symmetry classification, IR and Raman activities for 2H- and Td-WTe_2 structures. (A = active)

**Structure**	**2H-WTe_2**				**Td-WTe_2**			
**Mode**	Frequency (cm^{-1})	Classification	IR-active	Raman-active	Frequency (cm^{-1})	Classification	IR-active	Raman-active
**1**					9	A_1	A	A
**2**	28	E_{2g}		A	25	B_1	A	A
**3**	126	E_{1g}		A	28	A_2		A
**4**	183	A_{1g}		A	73	A_1	A	A
**5**	200	E_{2g}		A	85	B_1	A	A
**6**	201	E_{1u}	A		91	B_2	A	A
**7**	242	A_{2u}	A		95	A_2		A
**8**					115	A_2		A
**9**					116	B_2	A	A
**10**					119	A_1	A	A
**11**					119	A_2		A
**12**					121	B_2	A	A
**13**					122	B_1	A	A
**14**					127	B_1	A	A
**15**					130	B_1	A	A
**16**					133	A_1	A	A
**17**					134	A_1	A	A
**18**					137	A_1	A	A
**19**					138	B_1	A	A
**20**					159	B_2	A	A
**21**					159	A_2		A
**22**					164	B_1	A	A

**21**	167	A_2		A
**22**	168	B_2	A	A
**23**	168	A_1	A	A
**24**	181	B_1	A	A
**25**	182	A_1	A	A
**26**	216	A_1	A	A
**27**	218	A_1	A	A
**28**	220	B_1	A	A
**29**	239	B_1	A	A
**30**	239	A_1	A	A

The high resolution full range x-ray photoelectron spectroscopy (XPS) in Figure S3 is used to verify the bonding information for the exfoliated WTe_2. Td 3d (571.80eV) and W 4d (242.30eV) regions were selected based on the peaks not overlapping with the other elemental regions. XPS data verifies that Br is not incorporated in significant quantities into the WTe_2. Close observation at the Br 3p region (~188.3eV) and the Br 3d region (~68-70eV) shows that there are no Br peaks. Auger spectra in the higher binding energy region (not shown here) were also checked for Br peaks, and verified that Br was below detection limits.

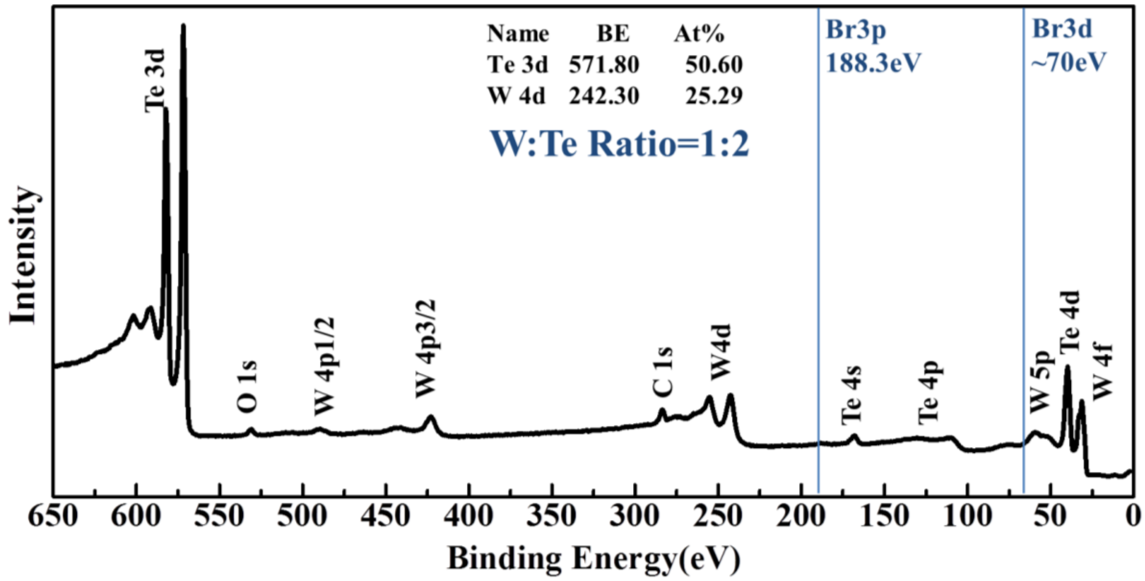

Figure S4. High-resolution XPS spectrum from a freshly exfoliated WTe_2 flake.

The analyzed XPS binding energies from freshly exfoliated WTe_2 and aged WTe_2 (5 day exposure to air) are listed in Table S4. There are two sets of Te 3d3/2 and 3d5/2 binding energies in the Te 3d data for the degraded sample, and the second set are obtained after normalization and subtraction of the fresh exfoliated WTe_2 Te 3d spectrum. The O 1s binding energy was processed similarly with the normalization and subtraction of the fresh exfoliated WTe_2 spectrum. The second set of binding energies at 586.60eV for Te 3d3/2, 576.10eV for Te 3d5/2 and 530.23eV in the O 1s region are referred to TeO_2 binding energies in the NIST XPS database.[4] Peak fitting of the W 4d spectra gives relatively similar binding energies for the W 4d3/2 and W 4d5/2 peaks in both samples.

Table S4. XPS analysis and peak fitting data for WTe_2 and degraded WTe_2 surface.

Region/Sample	Te 3d				O 1s		W 4d	
	Te 3d3/2		Te 3d5/2				W 4d3/2	W 4d5/2
	WTe_2	TeO_2	WTe_2	TeO_2	-	TeO_2		
**(1) Fresh exfoliated WTe_2 surface**	582.51	-	572.12	-	531.43	-	255.41	243.10
**(2) WTe_2 surface with 5 days exposure to air**	582.64	586.60	572.22	576.10	531.44	530.23	255.80	243.47

References

1. Kumar, A. & Ahluwalia, P. K. Semiconductor to metal transition in bilayer transition metals dichalcogenides MX2 (M = Mo, W; X = S, Se, Te). *Model. Simul. Mater. Sci. Eng.* **21,** 065015 (2013).

2. Kumar, A. & Ahluwalia, P. K. Electronic structure of transition metal dichalcogenides monolayers 1H-MX2 (M = Mo, W; X = S, Se, Te) from ab-initio theory: new direct band gap semiconductors. *Eur. Phys. J. B* **85,** 186 (2012).

3. Brown, B. E. The crystal structures of WTe2 and high-temperature MoTe2. *Acta Crystallogr.* **20,** 268–274 (1966).

4. Naumkin, A. V., Kraut-Vass, A., Gaarenstroom, S. W., & Powell, C. J., *NIST X-ray Photoelectron Spectroscopy (XPS) Database, Version 4.1*. (2012) Avalible at: http://srdata.nist.gov/xps/. (Access date: 7th January 2015)
